# Plasticity in the Sensitivity to Light in Aging: Decreased Non-visual Impact of Light on Cognitive Brain Activity in Older Individuals but No Impact of Lens Replacement

**DOI:** 10.3389/fphys.2018.01557

**Published:** 2018-11-06

**Authors:** Véronique Daneault, Marie Dumont, Éric Massé, Pierre Forcier, Arnaud Boré, Jean-Marc Lina, Julien Doyon, Gilles Vandewalle, Julie Carrier

**Affiliations:** ^1^Functional Neuroimaging Unit, University of Montreal Geriatric Institute, Montreal, QC, Canada; ^2^Center for Advanced Research in Sleep Medicine, CIUSSS-NÎM – Hôpital du Sacré-Cœur de Montréal, Montreal, QC, Canada; ^3^Department of Psychology, University of Montreal, Montreal, QC, Canada; ^4^Department of Psychiatry, University of Montreal, Montreal, QC, Canada; ^5^École d’Optométrie, University of Montreal, Montreal, QC, Canada; ^6^Génie Électrique, École de technologie supérieure, Montreal, QC, Canada; ^7^Centre de Recherches Mathématiques, Université de Montréal, Montreal, QC, Canada; ^8^GIGA-Institute, Cyclotron Research Centre–In Vivo Imaging, University of Liège, Liège, Belgium

**Keywords:** aging, cognition, light, circadian, melanopsin, non-visual impact of light, lens

## Abstract

Beyond its essential visual role, light, and particularly blue light, has numerous non-visual effects, including stimulating cognitive functions and alertness. Non-visual effects of light may decrease with aging and contribute to cognitive and sleepiness complaints in aging. However, both the brain and the eye profoundly change in aging. Whether the stimulating effects light on cognitive brain functions varies in aging and how ocular changes may be involved is not established. We compared the impact of blue and orange lights on non-visual cognitive brain activity in younger (23.6 ± 2.5 years), and older individuals with their natural lenses (NL; 66.7 ± 5.1 years) or with intraocular lens (IOL) replacement following cataract surgery (69.6 ± 4.9 years). Analyses reveal that blue light modulates executive brain responses in both young and older individuals. Light effects were, however, stronger in young individuals including in the hippocampus and frontal and cingular cortices. Light effects did not significantly differ between older-IOL and older-NL while regression analyses indicated that differential brain engagement was not underlying age-related differences in light effects. These findings show that, although its impact decreases, light can stimulate cognitive brain activity in aging. Since lens replacement did not affect light impact, the brain seems to adapt to the progressive decrease in retinal light exposure in aging.

## Introduction

Sleepiness and cognitive complains are common in older people. Up to 30% of community-dwelling older individuals present excessive daytime sleepiness (Foley et al., 2004; Nakakubo et al., 2016), which is not only associated with current cognitive impairments but also with increased risks fordeveloping cognitive decline (Merlino et al., 2010; Elwood et al., 2011). Sleepiness is also highly prevalent in most age-related neurological disorders (Merlino et al., 2010).

Light exposure can directly improve alertness and increase performance on several cognitive tasks in young individuals (for review Vandewalle et al., 2009). This impact is mediated through a “non-visual” (or “non-image-forming”) photoreception system that depends heavily on melanopsin-expressing retinal ganglion cells (mRGCs). These cells detect variations in ambient irradiance and are more sensitive to blue light (460–480 nm; LeGates et al., 2014). The non-visual impacts of light are therefore stronger when using blue or blue-enriched light compared to longer wavelengths (Vandewalle et al., 2009; Chellappa et al., 2011).

A good management of the light environment contributes to healthy aging notably with an improvement of alertness and cognition (Fetveit et al., 2003; Riemersma-van der Lek et al., 2008; Munch et al., 2011; Sander et al., 2015). However, aging may be associated with a reduction in the non-visual response to light likely through changes occurring at the level of the brain but also potentially at the level of the eye (Herljevic et al., 2005; Sletten et al., 2009; Daneault et al., 2012, 2014; Rukmini et al., 2017). Aging is indeed associated with crystalline lens yellowing and senile miosis (i.e., pupil size reduction), which reduce the amount of light, and particularly blue light, that reaches the retina. Whether these age-related eye modifications affect the non-visual effect of light on brain function is unknown. This question is not straightforward, because the aging brain may adapt, at least in part, to chronically decreased light reaching the retina, as was reported for younger individuals (Rufiange et al., 2007; Turner and Mainster, 2008; Gimenez et al., 2014).

In our aging societies, a full understanding of how light stimulates brain activity and why its impact would change over the lifespan is crucial to establish as an easy aids for alertness and cognition in aging.

Intraocular lens (IOL) replacement performed as part of cataract surgery increases importantly the amount of (blue) light transmission and restores a ∼20 year old clear lens (Turner et al., 2010). IOL provides therefore a unique human *in vivo* model to not only to evaluate the impact of lens yellowing on the non-visual impact of light on cognitive brain activity in aging, but also to assess potential brain adaptations in aging. If IOL was to have a limited effect on light impact despite the increased retinal illumination, it would imply that the aging brain adapts to lifetime changes in ambient lighting. However, to date, very few studies have assessed whether IOL replacement fosters the effects of light on non-visual functions in the elderly.

Functional magnetic resonance imaging (fMRI) has proven to be very sensitive to assess the impact of light quality on cognition (Vandewalle et al., 2009; Gaggioni et al., 2014). Variations in light impact with time-of-day and sleep loss were demonstrated using fMRI when behavioral measure were insensitive to such subtle differences (Vandewalle et al., 2011). These neuroimaging studies have indicated that, during early adulthood, the non-visual light signaling affects brain activity in alertness-related subcortical structures (brainstem, hypothalamus, thalamus) and limbic areas (amygdala, hippocampus) as well as in task-specific cortical areas (Vandewalle et al., 2009). Aging was reported to decrease the impact of light on brain activity (Daneault et al., 2014), but the protocol did not allow to conclusively separating visual and non-visual impact of light and did not address the potential role of senile miosis and lens yellowing.

Here, we first aimed to establish whether the non-visual impact of light on ongoing cognitive brain activity is reduced in aging. Second, we sought to determine whether lens yellowing and pupil miosis contribute to this decrease. We compared the effects of blue (480 nm) and orange (620 nm) monochromatic light on fMRI executive brain responses to auditory cognitive tasks in young healthy participants and in older healthy individuals either with their natural lens (older-NL), or with IOL replacement (older-IOL). We predicted that, compared to young subjects, elderly participants will show reduced effects of light on ongoing non-visual brain activity. We further postulated that the aging brain will not fully “adapt” to lens yellowing, such that, compared to older-NL individuals, older-IOL participants would show stronger non-visual effects of light on cognitive brain activity.

## Materials and Methods

This study received institutional ethics approval from the Comité d’éthique de la recherche vieillissement-neuroimagerie du CIUSSS du Centre-Sud-de-l’île-de-Montréal (CER VN du CIUSSS-CSMTL). Participants provided a written informed consent after detailed verbal and written information about the study. They received financial compensation for their participation.

### Participants

Thirty-eight healthy participants completed the study (Table 1): 14 younger (23.6 ± 2.5 years), 12 older-NL (66.7 ± 5.1 years), and 12 older-IOL (69.6 ± 4.9 years) participants. Exclusion criteria were as follows: sleep duration < 6.5 h or > 9 h; signs of mild cognitive impairment as indicated by MOCA (Nasreddine et al., 2005) (<26), anxiety, depression; body mass index (BMI) ≥ 28; smoking; high caffeine (>4 doses/day) or alcohol (>14 units/week) consumption; left dominant hand; medication affecting the central nervous system or sleep; night shift work during the past year; transmeridian travel during the past 3 months; history of psychiatric or sleep disorders. An extensive optometrist examination allowed ruling out ocular problems. The examination included review of ophthalmologic history, verification of visual acuity, binocular vision, color vision, and refraction, together with verification of healthy eye, optic nerve, central and peripheral retina, and assessment of intra-ocular pressure. Crystalline yellowing was assessed using the Lens Opacities Classification System, version III (LOCS III; Chylack et al., 1993). Participants were excluded for ophthalmologic reasons in case of color blindness, high ocular pressure, abnormal binocular vision, eye/retina/optic nerve disease (e.g., glaucoma). As indicated in Table 1, six participants in the IOL had ultraviolet (UV) only lens and six blue blocking lens which filter shorter wavelength light in addition to UV light. The six participants with UV only lenses and with blue blocking lenses did not differ in terms of any of the variables listed in Table 1 (*p* > 0.3).

**Table 1:** Participants’ characteristics (mean ± SD).

	Younger participants (Y) (*n* = 14)	Older participants with lens replacement (IOL) (*n* = 12)	Older participants with their natural lens (NL) (*n* = 12)	*P*
Age	23.6 ± 2.5	69.6 ± 4.9	66.7 ± 5.1
Laterality (right-handed)	14	12	12
Sex	9W/5M	10W/2M	7W/5M	0.39
Body mass index (BMI)	22.5 ± 2.9	24.3 ± 2.9	22.8 ± 2.8	0.27
Depression score (BDI-II)	1.3 ± 1.7	2.9 ± 4.1	4 ± 4.7	0.17
Anxiety score (BAI)	2 ± 1.7	3 ± 3	2.3 ± 2.8	0.58
Daytime Sleepiness (ESS)	4.7 ± 3.6	4.1 ± 2.9	4.7 ± 4.3	0.89
Sleep disturbance score (PSQI)	2.3 ± 1.3	3.3 ± 3.3	2.8 ± 2.4	0.59
Chronotype score (MEQ)	50.2 ± 7	56.9 ± 12.3	64.7 ± 9.3	0.002^∗^
				Y < IOL and NL
Years of education	15.7 ± 3.2	14 ± 1.8	15 ± 2.9	0.29
Photoperiod	13.5 ± 3.1	13 ± 2	12 ± 2.3	0.36
Bedtime prior to experiment	23:43 ± 0:38	23:19 ± 0:52	22:44 ± 1:04	0.02^∗^
				NL < IOL and Y
Wake time prior to experiment	7:59 ± 0:36	7:13 ± 0:55	7:06 ± 0:54	0.02^∗^
				Y > IOL & NL
Total sleep time prior to experiment (h:min)	7:03 ± 0:34	6:57 ± 0:52	7:12 ± 1:04	0.79
Volume level of auditory stimuli in fMRI (arbitrary units)	–1135 ± 287	–1288 ± 171	–1224 ± 227	0.19
LOCS-III	1 ± 0	1 ± 0	1.5 ± 0.8	0.03
				NL > IOL and Y
Type of intraocular lens		6 blue-blocking	
Time lag between experiment and eye surgery (years) – mean value for right and left eye		/6 UV-clear		
blue-blocking		4.9 ± 2.4		
UV-clear		5 ± 3.4		0.98


*BAI, Beck Anxiety Inventory; BDI-II, Beck Depression Inventory-II; LOCS-III, Lens Opacities Classification System III; MEQ, Morningness–Eveningness Questionnaire; PSQI, Pittsburgh Sleep Quality Index; ESS, Epworth Sleepiness Scale.*

### Experimental Protocol

Experiments were performed from September 2014 to December 2015 (Table 1), and the day length (i.e., dawn to dusk period) at the time of the experiment was similar in the three groups of participants. At least 1 week prior to the experiment, participants were trained and habituated to the MRI environment and tasks. Participants adopted an 8-h regular sleep schedule (bedtime and wake time within 30 min of their habitual schedule) for 7 days prior to the experiment [compliance verified by actigraphy (Actiwatch-Spectrum; Respironics, OR)]. Actigraphy data indicate no significant difference between sleep duration during the night just preceding the experiment and mean sleep duration during the six prior nights supporting that no partial sleep deprivation was induced by anticipation of the experiment [main effect of night (*F* = 0.98; *P* = 0.43); main effect of group (=0.39; *P* = 0.68); night × group interaction (*F* = 0.61; *P* = 0.81)]. Volunteers were requested to refrain from caffeine and alcohol consumption for 24 and 48 h, respectively, prior to the experiment.

**FIGURE 1:**
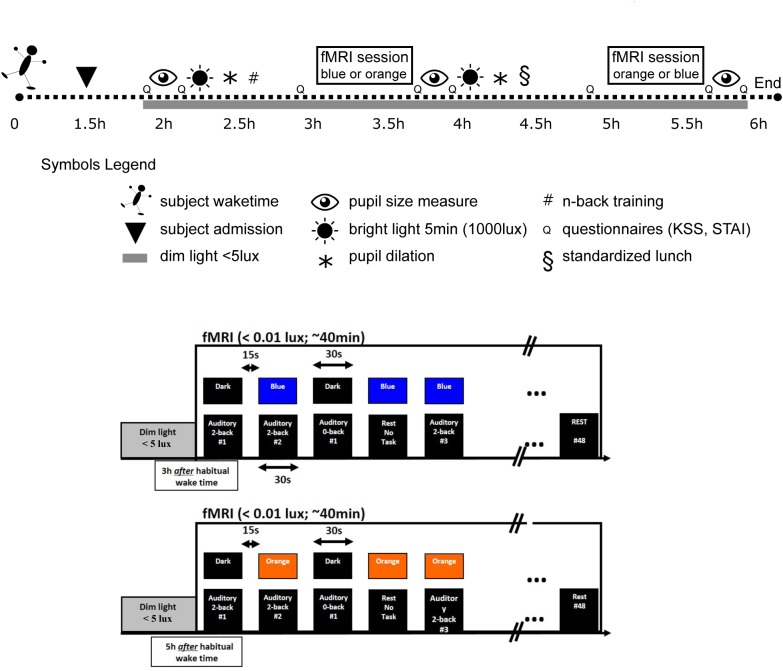
Experimental protocol. Upper panel: participants were maintained in dim light after admission (1.5 h after their habitual waketime). They completed two fMRI sessions under light exposure in a counterbalanced order (blue light 480 nm, 3 × 10ˆ13 ph/cm/s^2^ – orange light 620 nm, 3 × 10ˆ13 ph/cm/s^2^). The total duration of each session was 40 min during which participants performed an auditory n-back task. Upon arrival (baseline) and after each fMRI session, the pupils were measured. Pupils were dilated before, and right after, the first fMRI session (e.g., before second fMRI session). Subjective sleepiness and anxiety levels were also collected. Participants were exposed for 5 min to a bright polychromatic white light (1000 lux) before each fMRI session. Lower panels: within an fMRI session (i.e., including either blue or orange light), light was administered in block of 30 s and coincided with the 30 s task blocks (either 0b or 2b) or were administered alone. All blocks were separated by at least 15 s in complete darkness without task, which was either followed by a 30 s light exposure or a 30 s period in darkness. A total of 48 blocks of auditory 0b task (*n* = 18), 2b task (*n* = 20), and rest under light exposure (*n* = 10) were performed in each fMRI session. Half of n-back task blocks were executed under light exposure and the other half in darkness.

**FIGURE 2:**
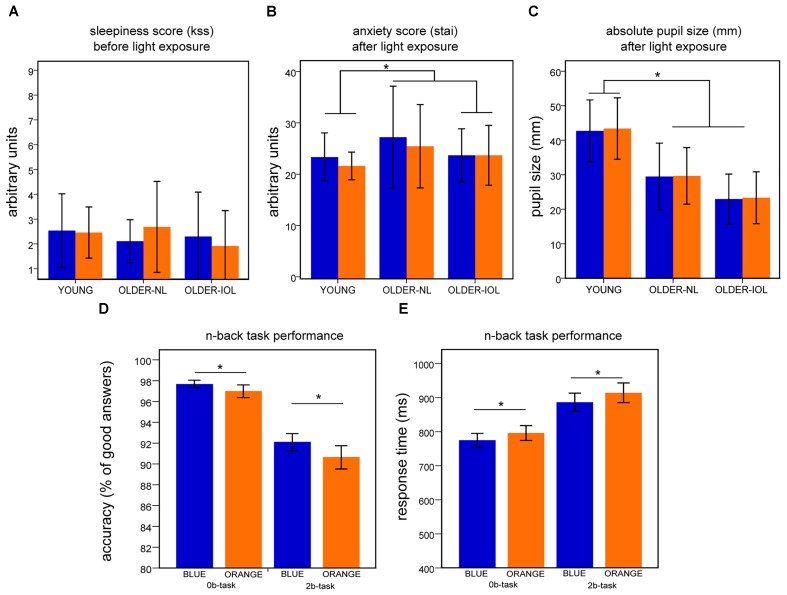
Behavioral results. For each panel **(A–E)**, results represent mean ± standard error of the mean (SEM) for both light conditions. **(A)** Score on the Karolinska (KSS) sleepiness scale before the fMRI session. **(B)** Score on the State Anxiety questionnaire (STAI-S) after each fMRI session. **(C)** Dilated absolute pupil size (mm) after each fMRI session. **(D)** Accuracy on the 2b and 0b tasks when all subjects are pooled together. **(E)** Response time to 2b and 0b tasks when all subjects are pooled together;^∗^ indicates significant differences between groups or light conditions.

The experiment was planned according to individual wake up time. Participants arrived at the laboratory 1.5 h after their habitual wake time and were maintained in dim light (<5 lux) thereafter (Figure 1). Two fMRI sessions were performed 3 and 5 h after the habitual wake time, during which participants were exposed to either blue or orange monochromatic light (counterbalanced order). To standardize previous light exposure, subjects were exposed for 5 min to a bright polychromatic white light (1000 lux) 1 h before each fMRI session and pupil constriction was pharmacologically inhibited with two drops of Tropicamide administered 30 min before each fMRI session. Pupil dilation was included to avoid the known pupil constriction differences between light condition (Cajochen et al., 2005) that would have bias light condition comparison. Importantly, pupil dilation does not remove age-related pupil size differences (as seen in Figure 2C). Each fMRI session lasted 40 min and there was a gap of 1 h 20 min between each session. Pupil size was measured three times: at baseline (i.e., prior to pupil dilation), and after the first and second fMRI session. Subjective sleepiness [Karolinska sleepiness scale (KSS)] (Akerstedt and Gillberg, 1990) and anxiety state [State Anxiety Questionnaire (STAI-S)] (Spielberger and Vagg, 1984) were evaluated five times across the protocol (baseline, before and after each fMRI session).

### Auditory Cognitive Tasks

During fMRI sessions, subjects performed a “0-back” (0b) letter detection task and a 2-back (2b) working memory task (Akerstedt and Gillberg, 1990). These tasks were used because they induce well characterized and reproducible brain responses (Owen et al., 2005) and were used to reliably probe the impact of light on cognitive brain activity. Importantly, we chose to include them in the auditory modality to ascertain that non-visual aspects of brain function would be affected by light exposure. Auditory cues were given to specify block type, either 0b or 2b, before each block. In the 0b task, participants indicated whether the consonant was a “K” or not using a “yes” or “no” keypress. In the 2b task, volunteers stated whether or not each consonant was identical to the letter presented two items earlier. Brain activity related to the 0b task was subtracted from brain activity associated with the 2b task to isolate the executive component of the 2b task and to control for potential baseline changes (Owen et al., 2005). N-back blocks lasted 30 s and included 14 auditory stimuli. Stimuli lasted 500 ms, inter-stimulus intervals were 2100 ms and series of stimuli included 30% of hits. Stimuli were produced using COGENT 2000^1^ in MATLAB (Mathworks, MA) and transmitted using MR CONTROL amplifier and headphones (MR Confon, Germany). Before starting each fMRI session, volunteers set their own optimal volume level. All blocks of task were separated by at least 15 s of rest (i.e., no task).

Importantly, immediately following screening, participants were extensively trained to the auditory n-back tasks subsequently used in the MR scanner for the experiment. Training included 24 2b blocks (of 30 s) and 14 0b blocks. Participant had to reach 75% of correct responses at the end of training in order to be included (training #1–3). Participants were trained again the both tasks 1 h prior to the first fMRI session (training #4). Training aimed to reduce interindividual differences in performance in the MR scanner that could bias fMRI results.

### Light Exposure

Light was delivered from the light source to the participants’ eyes, in the MR scanner, using an optic fiber (Dolan-Jenner, Boxborough, MA, United States) projecting onto diffusors fixed on google designed for the purposes of such studies (light was placed at 5 cm from the glass and the diffusors permit a uniform illumination of most of the field of view). Polychromatic white light was produced using an LED light source (Sugar cube, Edmund optics, NJ) and filter using narrow band-passed filter to produce either blue [480 nm – full-width at half-maximum (FWHM): 10 nm) or orange (620 nm – FWHM: 10 nm] lights of equal photon densities at eye levels (3 × 10^13^ photons/cm^2^/s; blue light: 12.5 uW/cm^2^; orange light: 9.6 uW/cm^2^). Light wavelengths were selected to be equidistant from the peak sensitivity of the photopic visual system (550 nm; Rodieck, 1998), while blue light coincided with the peak sensitivity of the non-visual melanopsin-based photoreception (460–480 nm; LeGates et al., 2014).

Within an fMRI session (i.e., including either blue or orange light), light was administered in block of 30 s and coincided with the 30 s task blocks (either 0b or 2b) or were administered alone. All light blocks were separated by at least 15 s in complete darkness without task, which was either followed by a 30 s light exposure or a 30 s period in darkness.

### MRI and fMRI Data Acquisitions

MRI data were acquired using a 3 T MRI scanner (TIM-Trio, Siemens, Germany). Structural brain images acquired during the habituation session consisted of a T1-weighted 3D MP-RAGE sequence (TR 2300 ms, TE 2.94 ms, TI 900 ms, FOV 256 cm × 173 cm, matrix size 256 × 256 × 176, voxel size: 1 mm × 1 mm × 1 mm).

Functional MRI time series consisted of multislice T2^∗^-weighted images obtained with a gradient echo-planar sequence (EPI) (32 axial slices; voxel size: 3.4 mm × 3.4 mm × 3 mm with 30% gaps; matrix size 64 × 64 × 32; repetition time = 2000 ms; echo time = 30 ms; flip angle = 90°). Each fMRI session (∼40 min) included 10 blocks of 30 s with light alone (i.e., no task) and 38 blocks of tasks (0b/2b) in darkness or under light exposure (pseudorandom order). All blocks were separated by 15 s of quiet rest in darkness. Subjects completed 18 0b blocks (50% with light), 20 2b blocks (50% with light), and 10 blocks of light alone.

### Pupil Measurements

Eyes pictures were taken using a numeric camera (EOS-7D Canon Canada Inc.), equipped with 72 mm lens (EF-S, Canon; 15–85 mm; 1:3.5–5.6 IS USM). The camera was posted on a tripod, equipped with a 24 LED panel (ML240, Manfrotto; 8.4 lux at eye level). Subjects were installed on a chin rest with a standard ruler fixed to the upper horizontal bar. Pictures of the subject’s eyes were taken at a ∼40 cm. Width and height of pupil were computed using Gimp software (version 2.8.14^2^), by counting the number of pixels on the horizontal and vertical axes of the pupil area with respect to the number of pixel corresponding to the 1 cm of the ruler of each picture. Pupil size was computed as an ellipse surface (pi × ½ width × ½ height).

### Behavioral and Pupil Analysis

Statistical analyses of behavioral and pupil measures were carried out using SPSS (version 24, Chicago, IL, United States). Performance to the cognitive tasks during training [accuracy (% of correct responses)] and response time values (ms) were analyzed using a repeated measures ANOVAs with task type (2b; 0b) and training repetition as within subject measures, and group (young, older-IOL, older-NL), as between subject factor. Performance to the cognitive tasks in the MR scanner [accuracy (% of correct responses) and response time (ms) values] was analyzed using repeated measures ANOVAs with task type and light condition (blue, orange), as within subject measures, and group as between subject factor. Subjective sleepiness scores, anxiety values, and pupil measures after light exposure were analyses using repeated measures ANOVAs with light as the within-subject measure and group as the between-subject factor. *Post hoc* tests were performed with the Tukey (HSD) test (adjusted for multiple comparisons).

### fMRI Data Analysis

Data were analyzed using Statistical Parametric Mapping (SPM12^3^). Structural images were segmented and normalized using Dartel [which includes smoothing – 8-mm FWHM Gaussian kernel; normalization to Montreal Neurological Institute (MNI) space; Ashburner, 2007] to reduce deformation difference between age groups. FMRI data were normalized using structural image parameters.

Statistical analysis was conducted using a standard general linear model in two serial steps accounting for individual-level fixed effects and group-level random effects [high-pass filtering: 512 s; autoregressive (order 1) plus white noise model and a restricted maximum likelihood algorithm; Ashburner et al., 2017]. Both fMRI sessions were included in the same design matrix. Boxcar functions convolved with a canonical hemodynamic response function modeled each experimental condition with distinct regressors in each session (i.e., 0b in darkness, 2b in darkness, 0b with light, 2b with light, light alone). Separate stick functions convolved with the hemodynamic response modeled light-on and light-off, as the events may be associated with visual responses (of no interest in the present study) as well as error and non-response during 0b and 2b tasks.

Individual contrasts of interest consisted of (1) brain responses to executive tasks irrespective of light condition *[2-back blocks minus 0-back block (2b-0b) across all conditions]* and (2) effect of blue light *versus* orange light on brain responses to executive tasks *[(2b-0b)_blue_ - (2b-0b)_orange_].* These individual level fixed effects contrasts were then included in a random effects group-level analyses which consisted of a full factorial analyses with an unequal variance to identify the main effect of group, testing between group factors (younger, older-IOL, older-NL). To isolate which factor was driving the group differences, we further conducted one-sample or two-sample *t*-tests. Separate regression analyses were computed between brain responses to executive tasks *[(2b-0b)_blue_ - (2b-0b)_orange_]* and covariates: pupil size, task performance, and estimate of frontal/parietal brain responses (individual average of significant voxels in a 10 mm radius sphere around the peak group differences).

*t*-statistics maps had a threshold at *p* = 0.05 following a family wise error (FWE) correction over the whole brain volume [contrast of interest (1)] or on 10 mm spherical volumes around *a priori* locations of interest determined based on the literature in structures involved in the n-back tasks and working memory, arousal regulation, and salience detection or involved in non-image-forming effect of light in previous research.

## Results

### Pupil Size and Performance to the n-Back Task

There were no significant effects of light (*F* = 2.2; *p* > 0.15) or group (*F* = 1.4; *p* > 0.3) on subjective sleepiness, but a tendency toward a light-group interaction (*F* = 2.9; *p* = 0.07; Figure 2A). There was no significant effect of light (*F* = 2.5; *p* > 0.1), group (*F* = 1.4; *p* = 0.3), or light–group interaction on anxiety (*F* = 0.6; *p* > 0.5; Figure 2B). Despite pupil dilation, pupil size differed across groups [main effect of group (*F* = 19.5; *p* < 0.001)]: younger individuals had larger pupil than older participants (NL and IOL; *p* < 0.05) and older-IOL tended to have smaller pupil size compared to older-NL (*p* < 0.07). There was no main effect of light (*F* = 0.7; *p* = 0.4), as well as no group by prior light condition interaction for pupil size (*F* = 0.09; *p* = 0.9; Figure 2C).

All subject reached high performance to the two cognitive tasks during training (Supplementary Figure S1). Analyses of training performance showed that both groups learned the task and that older individuals were overall performing worse the younger individuals (Supplementary Figure S1). While in the MR scanner, however, no group differences were detected. All subjects showed high accuracy on the two cognitive tasks (>85%; Figure 2D). No main effect of age group or interaction between age group and light was observed for accuracy and reaction time (*F* < 1.1; *p* > 0.4). In contrast, a significant effect of light condition was observed (i.e., 0b and 2b together; *F* = 6.2; *p* < 0.02): performance (accuracy) was better under blue than under orange light (Figure 2D); for coherence with the fMRI analyses – below, only the blocks including light were considered here. This was also the case when considering only older individuals (*F* = 7.2; *p* < 0.013). A repeated measures ANOVA on reaction times revealed a significant main effect of the light condition (*F* = 51.2; *p* < 0.001; Figure 2E): speed was increased under blue as compared to orange light. The brief (30 s) exposure to blue light appears therefore to be sufficient to improve both accuracy and reaction times as compared to the orange light. Importantly however, following extensive training, no significant differences were detected between groups such that any group difference detected in fMRI data is unlikely to arise from a behavioral bias.

Aging, but not lens yellowing, modifies the impact of blue light on auditory cognitive brain responses.

**FIGURE 3:**
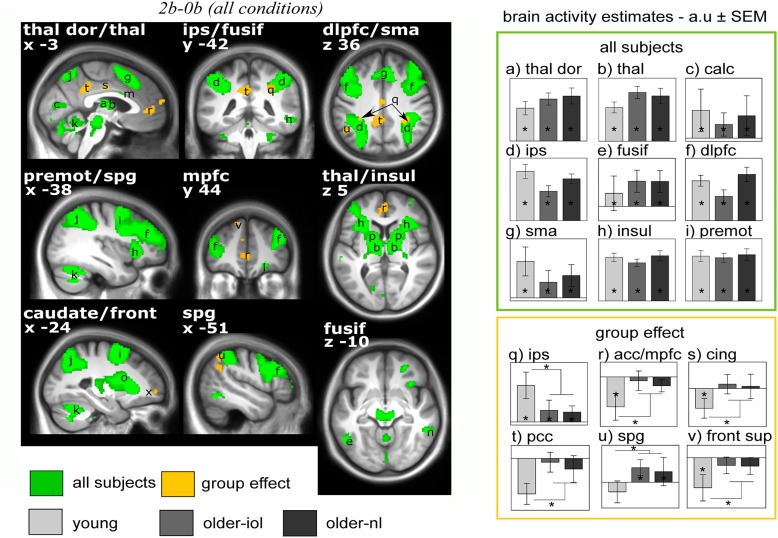
Brain responses to the n-back tasks [2b-0b] irrespective of the light conditions. Statistical results (*P* < 0.05 FWE-whole brain) overlay the mean structural image of all participants. Significant responses to the task common to all groups are displayed in green while group differences (older-nl and older-iol > younger; younger > older-nl and older-iol) are in yellow. See Table 2 for brain regions corresponding to the letters and abbreviations. Right panels show activity estimates (arbitrary unit – a.u. ± SEM) for each brain region. ^∗^ Significant group differences *P* < 0.05 corrected FWE-whole brain.

We first considered fMRI data irrespective of the light condition (i.e., including all block of 2b and 0b) and isolated executive brain activity by subtracting 0b brain responses from 2b responses *[2b-0b].* Executive responses across all three groups yielded typical activations in the prefrontal, parietal and cingulate cortices, thalamus, and putamen (Table 2 and Figure 3; Owen et al., 2005). We also observed expected age-related differences in executive brain responses *[2b-0b]* (Cabeza et al., 2004): compared to the young, older individuals (IOL and NL) presented lower activation in the intraparietal sulcus but higher activations in prefrontal, parietal, and cingulate cortices. Higher activations in older individuals likely reflect the recruitment of additional brain regions (“compensation”) to maintain performance comparable to that of the young (Esposito et al., 2009; Sambataro et al., 2010).

**Table 2:** Brain responses *[2b-0b across all conditions].*

Regions	*X*, *Y*, *Z*	*T*	*P*-value
**One-way Anova – brain activations for all subjects**			

Thalamus dorsal [a]	12 –6 10	11.31	<0.001
	–10 –6 10	9.87	<0.001
Thalamus [b]	14 –6 10	11.57	<0.001
	–12 –12 6	12.36	<0.001
Occipital (Ccalcarine) [c]	–8 –78 10	7.19	<0.001
	12 –70 14	6.81	<0.001
	4 –72 12	6.35	0.005
Intraparietal sulcus (IPS) [d]	–32 –43 51	6.33	<0.001
	32 –44 50	5.93	<0.001
	34 –48 48	10.10	<0.001
Fusiform [e]	–44 –60 –14	5.86	<0.001
Dorsolateral prefrontal cortex (DLPFC) [f]	44 36 32	9.07	<0.001
	–46 32 36	9.67	<0.001
Supplemental motor area (SMA) [g]	6 12 52	10.94	<0.001
	–4 14 50	13.89	<0.001
Insula/fronto-insular [h]	32 24 –2	17.45	<0.001
	–32 22 0	15.26	<0.001
	32 26 2	15.45	<0.001
	–28 28 2	11.52	<0.001
Motor/Ppremotor [i]	–44 6 34	15.31	<0.001
Superior parietal gyrus (SPG) [j]	–26 –58 –62	6.34	<0.001
	28 58 60	8.71	<0.001
Cerebellum [k]	28 –60 –30	14.99	<0.001
	30 –66 –48	13.52	<0.001
	–32 –54 –30	13.15	<0.001
Orbiofrontal Ccortex (OFC) [l]	24 44 –14	9.08	<0.001
Cingular [m]	4 8 28	8.11	<0.001
Temporal Iinferior [n]	–52 –60 –10	9.08	<0.001
	56 –46 –10	8.63	<0.001
Temporal Ssuperior	–58 –24 2	6.03	0.01
Caudate [o]	16 0 10	12.16	<0.001
	–12 2 6	11.13	<0.001
Putamen [p]	28 20 2	14.68	<0.001
	–26 14 4	10.43	<0.001

**Young > Older-NL and Older-IOL**			

IntraPparietal Ssulcus (IPS) [q]	30 –40 36	8.19	<0.001
	–28 –40 36	6.20	<0.004

**Older-NL and Older-IOL > Young**			

ACC/MPFC [r]	0 48 2	6.53	<0.004
	–10 46 0	6.34	<0.008
Mid-cingular [s]	–2 –14 36	6.81	<0.002
Precuneus (PCC) [t]	–2 –44 32	7.32	<0.001
	–6 –36 38	6.28	<0.002
Parietal superior and posterior [u]	–50 –56 48	6.19	<0.009
	–38 –68 40	6.14	<0.02
Frontal superior [v]	–12 44 48	6.27	<0.008
Frontal inferior [x]	–20 54 –2	7.05	<0.001
Angular gyrus	–46 –54 32	6.88	<0.002


*Brain responses to the n-back task [2b-0b] irrespective of light condition. Letters between [ ] correspond to letters of Figure 3. *P* < 0.05 values corrected for multiple comparisons over the entire brain volume (family wise error approach).*

**FIGURE 4:**
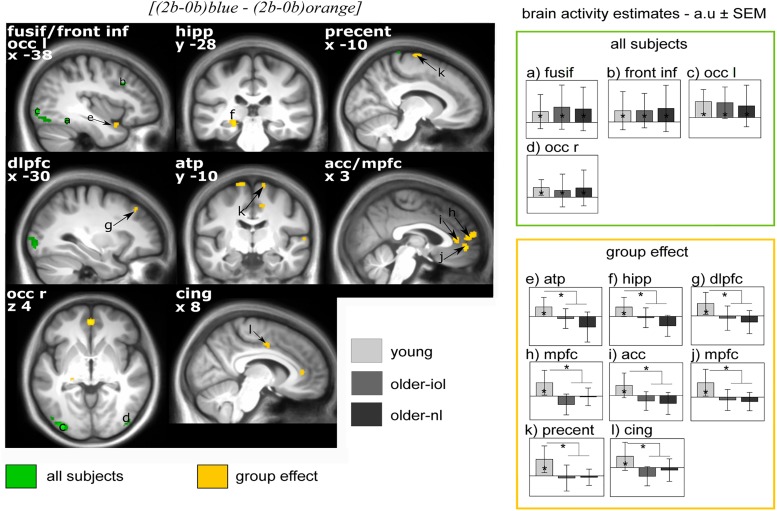
Effect of [(2b-0b)blue – (2b-0b)orange] on brain responses in young, older-nl, and older-iol subjects. Statistical results (*P* < 0.001 uncorrected) overlay the mean structural image of all participants. Responses to light are displayed in green for all subjects pooled together, whereas group differences (young > older-nl and older-iol) are displayed in yellow. Right panels show activity estimates (a.u. ± SEM) for each brain region. See Table 3 for brain regions corresponding to the letters and abbreviations. ^∗^ Significant group differences *P* < 0.05 corrected for multiple comparisons over small volumes of interest taken *a priori* location based on the literature.

We then evaluated the difference between light conditions, irrespective of groups: executive brain responses *[(2b-0b)_blue_ - (2b-0b)_orange_]* were higher under blue than under orange light in four brain regions (Figure 4), namely, the fusiform gyrus, inferior frontal cortex, left and right lateral occipital cortex. Brain activity estimates show increased executive responses in these regions under blue, as compared to orange light. Critically, multiple significant group differences were also detected in the comparison of the impact of the blue vs. orange light exposure on executive brain responses *[(2b-0b)_blue_ - (2b-0b)_orange_]:* in the anterior temporal pole (ATP), the left hippocampus, the dorsolateral and median prefrontal gyrus [dorsolateral prefrontal cortex (DLPFC), MPFC], the middle and anterior cingulate cortex (mid-cingular, ACC), as well as the precentral area (Figure 4). Inspection of activity estimates revealed that, in all these brain regions, younger individuals presented higher executive brain activation under blue vs. orange light, as compared to the two groups of older individuals. Two by two comparisons between groups confirmed that executive responses under blue vs. orange monochromatic light were high in younger vs. older-NL (see ^∗^ in Table 3) and in younger vs. older-IOL groups (see #; Table 3). Importantly, there were no significant differences between the two groups of older individuals, suggesting that clear ocular lenses (older-IOL participants) did not significantly modify the impact of light compared to the less transparent lenses (older-NL group). In fact, in the areas showing significant group differences, only the young group showed significantly higher activations under blue vs. orange monochromatic light (see §; Table 3).

**Table 3:** Brain responses for *[(2b-0b)_blue_*- *(2b-0b)_orange_]*.

Regions	*X*, *Y*, *Z*	*T*	*P*-value
**Common group activations**			

Fusiform gyrus [a]	36 –46 –18	4.06	≤0.001
Frontal inferior cortex [b]	–38 18 34	3.79	≤0.005
Occipital left (lateral) [c]	–34 –90 –4	5.92	≤0.02
	–48 –78 –4	3.72	≤0.005
Occipital right [d]	46 –82 –2	3.74	≤0.005
	32 –90 22	3.48	≤0.009

**Group differences**			

Anterior temporal pole [e]	– -36 8 -36–26^∗∘^	9.17	≤0.008
	– -50 4 – -22^∗^		
Hippocampus [f]	– -24 – -28 – -10^∗^#§°	11.31	≤0.002
DLPFC [j]	– -32 32 42^∗^#§°	9.70	≤0.005
MPFC [h,j]	– -2 52 – -4^∗^#§°	11.91	≤0.001
	2 52 – -4^∗^#§°	10.93	≤0.001
	2 56 8*#§°	9.48	≤0.005
ACC [i]	6 38 8*#§	10.42	≤0.003
Precentral gyrus [k]	40 – -18 66^∗^#§°	11.24	≤0.002
	– -14 – -10 76#§°	11.13	≤0.01
	14 – -8 72#§°	10.30	≤0.01
Mid cingular gyrus [l]	10 – -4 46#°	8.56	0.03


*Brain responses to the n-back task [(2b-0b)blue – (2b-0b)orange] according to light condition. Letters between [ ] correspond to letters of Figure 4. *P* < 0.05 values corrected for multiple comparisons over small volumes of interest taken a priori location based on the literature. In the group differences section, significant results are represented as ^∗^(young vs. older-nl); #(young vs. older-iol); §(young);° (with frontal and parietal areas as covariates).*

Pupil size, but not brain task engagement, may contribute to age-related differences in executive responses under blue light.

We investigated whether age-related differences in the impact of blue and orange monochromatic light exposure on executive brain responses were related to other group differences we detected. When using dilated pupil size (average of both post fMRI measurements) as a covariate, most group differences were attenuated. It could suggest that group difference in pupil size explains part of the group difference in the impact of blue vs. orange light on ongoing brain activity. However, pupil size is highly correlated to age in our sample (*r* = -0.74; *p* < 0.001), it is thus not possible to tease apart the individual contributions of pupil size and aging in our analyses.

One could also argue that light cannot be as impactful on brain activity in older individuals because those individuals are already more engaged in the task, as indicated by the higher executive brain responses observed in both older groups irrespective of the light condition (cf. Figure 3). To address this issue, we used as a covariate the individual estimates of executive brain responses in the prefrontal and parietal areas, which stand as the most typical regions recruited in an n-back task (Owen et al., 2005). Using these brain activity estimates as covariate did not affect the group differences in the impact of light, indicating that the age-related brain compensations are unlikely to significantly contribute to the differential impact of the light conditions on executive brain responses (see Table 3).

## Discussion

Light exposure could be a simple and easy mean to improve alertness and cognition in aging. The stimulating impact of light is, however, likely to change in aging potentially because of decreases in lens transmittance and/or pupil. Here, we assessed the direct effects of light on vigilance and cognition in healthy younger and older individuals using a sensitive neuroimaging approach. Crucially, a sample of older with IOL following cataract surgery was included as a unique human model to test the role of lens yellowing in age-related changes in the non-visual impact of light. Here, we provide compelling evidence that light can improve executive performance and its associated brain activity in healthy older individuals. We demonstrate, however, that the stimulating effect of light on ongoing cognitive brain activity is reduced in older individuals indicating that environmental light conditions should be adapted for older individuals to optimize their cognition. Surprisingly, lens replacement which enhances the amount of blue light reaching the retina did not increase brain sensitivity to light, suggesting that the older brain adapts to lens yellowing across time.

As compared with longer wavelength light, which triggers little mRGC phototransduction, shorter wavelength light, geared toward mRGC (LeGates et al., 2014) and delivered during the day, significantly increased brain responses to the executive auditory task in occipital and frontal areas in both younger and older individuals (cf. Figure 3). This is reminiscent of a previous study, contrasting only blue monochromatic light and darkness right after habitual bed time, and pointing to an effect of blue light in occipital areas in older individuals (Daneault et al., 2014). This occipital recruitment may be related to visual photoreception and to the visual role of mRGC (Ecker et al., 2010). Yet, our fMRI analysis could only isolate brain responses to executive tasks and the impact that blue or orange light had on these responses. The occipital recruitment is therefore also likely to be related to the ongoing auditory rather than to a visual response alone. The fact that prefrontal activity was also stimulated in older individuals strongly suggests that light can affect cognitive brain activity at all ages. This statement is further supported by behavioral measures. Repeated 30 s exposures to blue-containing light were indeed sufficient to improve performance and reaction times as compared with orange light exposure. This observation contrasts with previous fMRI studies which failed to find any behavioral impact of short blue light exposures (Vandewalle et al., 2009, 2011; Chellappa et al., 2014; Daneault et al., 2014). Assuming that fMRI is usually more sensitive than behavioral measures, we believe that these previous studies were underpowered to detect a relatively weak effect of light. The present study conducted during the morning hours, together with similar previous fMRI experiments conducted at night and during the day (Vandewalle et al., 2011; Chellappa et al., 2014; Daneault et al., 2014), unravel the brain mechanisms associated with the established non-visual effect of light on cognitive performance reported using longer duration exposures (Cajochen et al., 2005; Lockley et al., 2006).

Even if light can affect non-visual activity of the aged brain, our data point toward important age-related reductions in this effect. The usual light-induced intensification in ongoing cognitive brain activity was reduced with aging in frontal and cingular areas, involved in executive function and in attentional/emotional regulation (Womelsdorf et al., 2014), respectively, as well as in the hippocampus, which is most associated with long-term memory (Lech and Suchan, 2013). In these areas, group differences were driven by young individuals who were significantly influenced by blue light, confirming previous reports (Vandewalle et al., 2009; Daneault et al., 2014; Hung et al., 2017), while it was not the case in the older groups. Importantly, all experiments were carried out according to individual sleep-wake schedule supporting that between group differences in chronotype or in endogenous circadian timing (Kawinska et al., 2005; Mongrain et al., 2008) is unlikely to have contributed to our findings.

While in fMRI, older individuals did not perform worse than younger ones. This is likely to results from the extensive training preceding brain activity recordings. It may also be because (blue) light stimulation reduces group differences. Despite comparable performance, we find age-related differences in brain responses to executive tasks, which are unrelated to the light conditions; this is in line with more brain engagement or compensation in our sample of older individuals that allows to reach or maintain similar performance than younger individuals (Schneider-Garces et al., 2010). We previously reported that light was more potent in stimulating cognitive brain activity in more difficult conditions (Vandewalle et al., 2011). We therefore hypothesized that age-related reduction in the non-visual impact of light was potentially due to a greater task engagement at older age; if the older brain is already importantly recruited in a difficult task, light can increase recruitment much further. The present study does not support this hypothesis since (1) it included a 0b condition which controlled for baseline difference in brain activity and (2) because estimations of frontal and parietal task engagement was not underlying the age difference in the impact of light.

Alternatively, age-related differences in the non-visual effect of light may arise from the progressive reduction of retinal illumination during the lifespan. Visual acuity is often decreasing at older age, but the amount of light reaching the retina is also lower because of reduced pupil size and yellowing of the lens (Turner and Mainster, 2008). The pupil size was indeed smaller in our older healthy individuals even after the pupil dilation procedure. In contrast to frontal/parietal activity, in our data set, difference in pupil size appears to be related with the group differences in the impact of light. Given the high correlation between pupil size and age, we believe, however, that this is not clear evidence for an important role of retinal illumination in the non-visual effect of light.

Expert evaluation of lens transmittance on the LOCS scale may suggest that the significant difference in lens transmittance does not reflect an important lens yellowing in the NL group (cf. Table 1). An average value of 1.5 ± 0.8 (range from 1 to 3) in the NL group already corresponds to a significant yellowing of the lens. In contrast, lens replacement will provide lens that are as clear as those of young adults (Brondsted et al., 2013) as indicated by a value of 1 on the LOCS scale, corresponding to a floor effect. While the NL and IOL groups were both healthy and did not differ in age, they differed in the number of photons reaching their retina. The fact that the NL and IOL groups did not differ on the differential effects of blue vs. orange light on their ongoing cognitive brain activity suggests an adaptation of the brain to the ambient illumination. Future studies should evaluate older individuals with higher LOCS values to further test this hypothesis. This has been suggested by a study of younger individuals who wore blue-blocking glasses for two weeks (Gimenez et al., 2014). The adaptation we suspect in older age could result from a slow plasticity progressively accounting for the reduced retinal illumination in normal aging or from a faster plasticity (<56 months on average in our sample) that would account for the increased retinal illumination following lens replacement, or both. This further reinforces the idea that other age-related modifications than illumination underlie the decreased stimulating effect of light in aging, potentially including brain changes, a reduction in mRGC number (Lax et al., 2016; Esquiva et al., 2017), or a shift in the spectral sensibility to light as suggested when considering melatonin suppression in a small sample of older individuals (*n* = 8; Najjar et al., 2014).

Lens replacement has been reported to improve visual acuity, color perception, and dark adaptation (Shenshen et al., 2016). Interestingly, inconsistent effects on sleep and circadian characteristics have been reported. Improvements in subjective sleep quality and latency have been reported (Ayaki et al., 2013; Alexander et al., 2014), but these results were not always replicated (Brondsted et al., 2015; Zambrowski et al., 2017). Likewise, melatonin secretion was found to be higher or unchanged 3 weeks (Brondsted et al., 2015) or 1 month after surgery (Tanaka et al., 2010). These inconsistencies may be related to the different types of lenses that are implanted during cataract surgery, with around 50% of IOL blocking only UV, while the other 50% also blocks visible blue light to prevent macular degeneration. However, most studies failed to reveal significant differences between lens types as confirmed in a meta-analysis focusing on sleep quality (Erichsen et al., 2015). Our results support the notion that, at least for cognitive cerebral activation, a clearer lens does not improve non-visual brain sensitivity when including an equal proportion of both types of IOL. Investigating the potential impact of lens type on cognitive brain activity will require further investigation.

Many neurological disorders are more prevalent with aging. In most, if not all, of these age-related disorders, excessive daytime sleepiness is an important complaint/symptom which is closely associated with declining cognitive status (Merlino et al., 2010) and with lower quality of life, shorter life expectancy, higher medical costs, and more institutionalization (Elwood et al., 2011; Nakakubo et al., 2016). The use of controlled light exposure has been suggested to be beneficial in patients with Parkinson’s and Alzheimer’s diseases (Ancoli-Israel et al., 2003; Riemersma-van der Lek et al., 2008; Videnovic et al., 2017) such that light is a promising and easy mean to improve alertness, cognition, and sleep in both healthy and pathological aging (Ancoli-Israel et al., 2003; Fetveit et al., 2003; Riemersma-van der Lek et al., 2008; Munch et al., 2011; Hopkins et al., 2017). We demonstrate a reduction in the non-visual effect of light on cognitive brain functions but other tasks and other light wavelength compositions (including polychromatic white light), as well as other aspects of the broad spectrum of the non-visual effects of light, still need to be investigated thoroughly. Optimal light administration in aging cannot be solely based on findings in young individuals and should take into account the gradual plasticity in the sensitivity to light in aging.

## Author Contributions

VD acquired and analyzed the data, designed the experiments, and wrote the paper. MD designed the experiments and wrote the paper. ÉM acquired and analyzed the data. PF provided access to cataract patient and ophthalmologic expertise. JD, AB, and J-ML provided expertise for MRI data acquisition and analyses. GV and JC analyzed the data, designed the experiments, and wrote the paper.

## Conflict of Interest Statement

The authors declare that the research was conducted in the absence of any commercial or financial relationships that could be construed as a potential conflict of interest.

## Footnotes

1 http://www.vislab.ucl.ac.uk/Cogent

2 https://www.gimp.org/fr/

3 http://www.fil.ion.ucl.ac.uk/spm
