# Supplementary material for: Plasticity in the Sensitivity to Light in Aging: Decreased Non-visual Impact of Light on Cognitive Brain Activity in Older Individuals but No Impact of Lens Replacement
Source: Front Physiol. 2018 Nov 6;9:1557. doi: 10.3389/fphys.2018.01557 (PMC6232421; doi:10.3389/fphys.2018.01557)
Supplement: Supplementary file 1 [file Image_1.PDF]

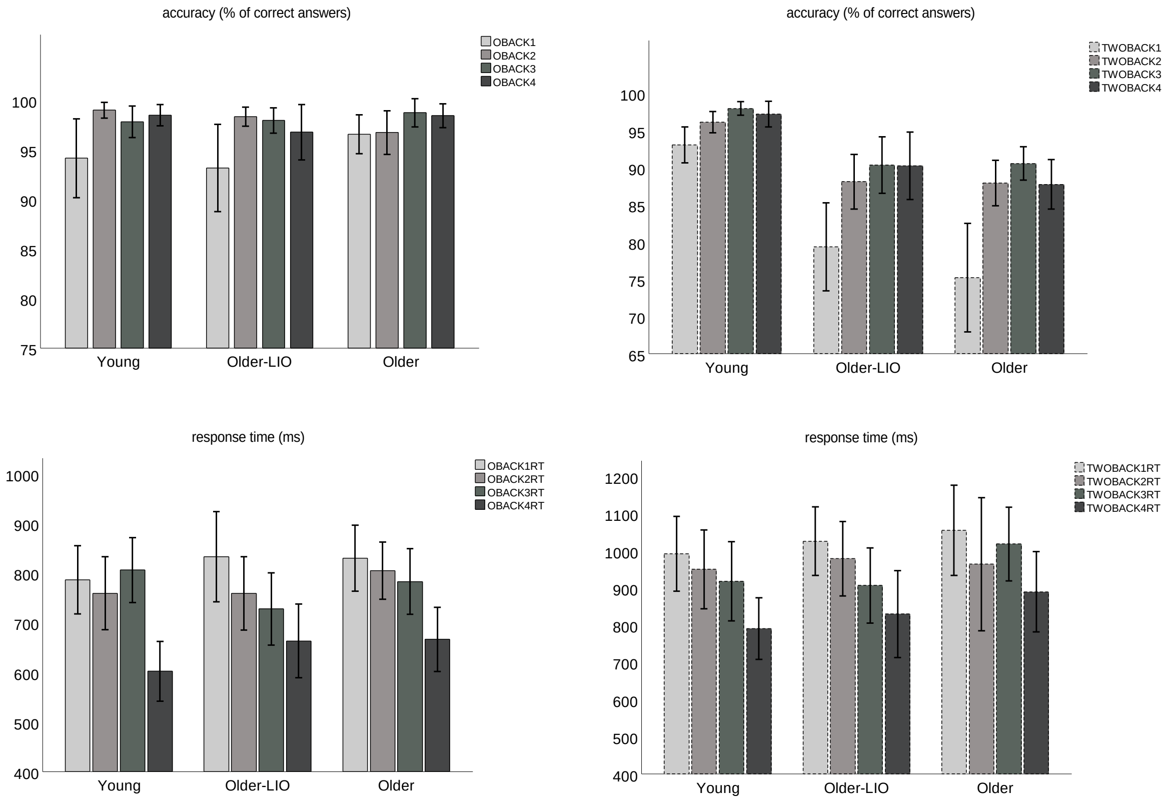

Supplementary Figure S1. Performance to the task during training.

Subjects were extensively trained to the tasks at screening (training 1-3) and 1h before entering the MR scanner (training 4). Each training includes 0b (0BACK) and 2b (TWOBACK) tasks. Training 1 and 2 included 17 blocks, 6 for the 0-back and 11 for the 2-back; training 3 and 4 included 8 blocks, 6 for the 2-back and 2 for the 0-back. Participant had to reach 75% of correct responses at the end of training 3 to be included.

Repeated measure ANOVA on accuracy with training (1-4) and tasks type (0b-2b) as within subject factors and group as between subject factor yielded a significant task type x training x group interaction ($F(2,35) = 12.45$, $p < 0.001$), a significant task type x training interaction ($F(3,105) = 11.5$, $p <0.001$), a main effect of task type ($F(1,35) = 103.02$, $p < 0.001$) and a main effect of training $F(3,105) = 33,07$, $p < 0.001$).

Repeated measure ANOVA on reaction times with training (1-4) and tasks type (0b-2b) as within subject factors and group as between subject factor yielded a significant main effect of task type ($F(1,35) = 121.62$, $p < 0.001$) and a main effect of training $F(3,105) = 34.25$, $p < 0.001$).
